# Age and Gender Disparities in Processed Meat Consumption During the COVID-19 Pandemic

**DOI:** 10.1093/geroni/igaf122.2305

**Published:** 2025-12-31

**Authors:** Lillie Monroe-Lord, Azam Ardekani, Phronie Jackson, Weitman Cassidy

**Affiliations:** University of the District of Columbia, Washington, District of Columbia, United States; University of the District of Columbia, Washington, District of Columbia, United States; University of the District of Columbia, Washington, District of Columbia, United States; University of the District of Columbia, Washington, District of Columbia, United States

## Abstract

The COVID-19 pandemic significantly altered dietary habits, including processed meat consumption, which holds critical health implications. This cross-sectional study utilized the Dietary Screening Tool (DST) to analyze age- and gender-related differences in processed meat consumption among 10,050 U.S. adults aged 40–100 years. Three age groups (40–60, 61–80, and 81–100 years) were compared using binary logistic regression to assess the odds of decreased processed meat consumption by age and gender. Results revealed significant disparities: women were 23.6% less likely than men to report reduced processed meat consumption. Participants aged 40–60 years were 47% more likely to reduce processed meat consumption compared to those aged 61–80 years and over twice as likely compared to those aged 81–100 years. The difference between the 61–80 and 81–100 age groups was not statistically significant. These findings suggest that younger adults, particularly those aged 40–60 years, were more likely to decrease processed meat consumption, possibly due to heightened health awareness during the pandemic. In contrast, women exhibited a lower likelihood of reduced processed meat consumption, underscoring potential gender-specific challenges in dietary habits. This study highlights the need for tailored dietary interventions addressing processed meat consumption. Public health strategies should focus on promoting healthier dietary patterns through targeted messaging for diverse demographic groups, particularly during public health crises.

